# Impact of Mattress Use on Sacral Interface Pressure in Community-Dwelling Older Adults

**DOI:** 10.3390/geriatrics10040107

**Published:** 2025-08-06

**Authors:** Hye Young Lee, In Sun Jang, Jung Eun Hong, Je Hyun Kim, Seungmi Park

**Affiliations:** 1 College of Nursing & Research Institute of Nursing Science, Chungbuk National University, Cheongju 28644, Republic of Korea; hyoung28@chungbuk.ac.kr (H.Y.L.); n112003@naver.com (J.E.H.); 2 Department of Nursing, Korean Bible University, Seoul 01757, Republic of Korea; agape90@bible.ac.kr; 3 Cheongju Joo Chan Mi Visiting Nursing Center, Cheongju 28696, Republic of Korea; ss6743@naver.com

**Keywords:** pressure ulcer, pressure, aged, long-term care, beds

## Abstract

Background/Objectives: Pressure injuries are a significant concern among older adults, particularly in community-based long-term care settings where prolonged immobility is prevalent. This study aimed to identify factors influencing sacral interface pressure in community-dwelling older adults, with an emphasis on support surface usage and clinical risk indicators. Methods: A total of 210 participants aged 65 years and older, all receiving long-term care services in South Korea, were enrolled in this study. Sacral interface pressure was measured in the supine position using a portable pressure mapping device (Palm Q7). General characteristics, Braden Scale scores, Huhn Scale scores, and mattress usage were assessed. Data were analyzed using descriptive statistics, *t*-tests, chi-square tests, and logistic regression. Results: Mattress non-use was identified as the strongest predictor of elevated sacral interface pressure (OR = 6.71, *p* < 0.001), followed by Braden Scale scores indicating moderate risk (OR = 4.8, *p* = 0.006). Huhn Scale scores were not significantly associated with interface pressure. These results suggest that support surface quality and skin condition have a stronger impact on interface pressure than mobility-related risk factors. Conclusions: The findings highlight the importance of providing high-quality pressure-relieving mattresses and implementing standardized nursing assessments to reduce the risk of pressure injuries. Integrating smart technologies and expanding access to advanced support surfaces may aid in developing tailored preventive strategies for vulnerable older adults.

## 1. Introduction

Population aging is progressing rapidly worldwide, posing a critical challenge to healthcare systems and society as a whole [1]. According to the United Nations Department of Economic and Social Affairs (DESA) in the World Population Prospects 2024, the global population entered an aging society as of 2022, and is projected to become an aged society by 2039 and a super-aged society by 2070 [1]. In particular, the proportion of the population aged 80 and above is expected to rise from 19.8% in 2023 to 38.9% by 2100, placing increased pressure on healthcare systems, human resources, and medical expenditures [1].

South Korea is experiencing an even faster rate of aging than global trends, with the elderly population aged 65 and older surpassing 20% of the total population in December 2024—making it the second super-aged society in Asia after Japan [2]. As the number of older adults rapidly increases, so does the demand for healthcare and long-term care services. In response, the Korean government implemented the Long-Term Care Insurance (LTCI) system in 2008 [3]. This system provides community-based services (e.g., home care, home nursing, adult day care) and institutional services (e.g., nursing homes, long-term care hospitals) depending on the individual’s health status and level of care needs [3]. A large proportion of LTCI beneficiaries are at high risk for pressure injuries due to chronic illnesses associated with aging, reduced mobility, and prolonged bed rest [4,5].

Pressure injuries are defined as ischemic damage to localized tissue resulting from sustained mechanical pressure on specific body areas [6]. In bedridden positions such as the supine posture, pressure tends to concentrate on bony prominences like the occiput, scapula, sacrum, and heels, increasing the risk of pressure injuries [7]. The development of pressure injuries is multifactorial, involving health status, reduced mobility, nutritional deficits, and particularly, interface pressure between the body and the support surface—a key physiological mechanism [8,9]. They are among the most common skin issues in older adults, with prevalence reported in up to 70% of those aged 70 and above [10]. The prevalence of pressure injuries in long-term care facilities ranges from 16.7% to 43.3%, higher than that of hospitalized patients, which ranges from 4.3% to 28.8% [11].

Interface pressure refers to the mechanical pressure exerted between the skin and the support surface. When sustained or exceeding a critical threshold, it can impair local blood flow and lead to tissue necrosis [12].

Since interface pressure is affected by multiple factors such as posture, mobility, skin condition, and body weight, its quantitative assessment is essential for developing individualized nursing interventions for pressure injury prevention [13]. Previous studies on interface pressure have mostly focused on pressure measurements in specific body positions or used single mattress conditions in controlled environments. As such, research comprehensively examining the factors affecting interface pressure in the commonly maintained sacral posture within real-world clinical or community settings remains limited [14].

In South Korea, the LTCI system currently focuses primarily on human resources—such as home care workers and visiting care services—while support for pressure-relieving equipment remains limited [15]. Although pressure-relieving mattresses are included in LTCI benefit items, they are available only for rental, not purchase. Most rental mattresses are low-cost models that often generate noise, have poor durability, and cause user discomfort, making them unsuitable for long-term use [15,16]. This creates a substantial financial burden for older adults and their families, while the lack of quality standards and post-use management further reveals systemic limitations [17]. By contrast, countries like Japan and Germany have developed integrated systems such as TAIS (Technical Aids Information System), MDK (Medizinischer Dienst der Krankenversicherung) reviews, and the REHADAT (Rehabilitations-Datenbank) database to assess equipment appropriateness and provide nationwide information [18,19]. These examples emphasize the importance of establishing a comprehensive support system that includes not only nursing care but also preventive equipment provision.

To address this research gap, the present study was conducted among older adults living in the community, reflecting real-life environments and the accessibility of local resources. In particular, the study incorporated various types of mattresses actually used in community-based care settings, and comprehensively analyzed multiple clinical variables, including Braden Scale scores, fall risk, and mattress use.

Therefore, this study aims to identify the factors influencing sacral interface pressure in community-dwelling older adults and to provide foundational evidence for the development of tailored community-based nursing interventions and health policy improvements to reduce pressure injury risks.

## 2. Materials and Methods

### 2.1. Study Design

This study employed a correlational research design to measure sacral interface pressure in older adults aged 65 and above receiving long-term care services in South Korea and to identify factors influencing pressure levels for the purpose of developing effective pressure injury prevention strategies.

This study employed a correlational research design to measure sacral interface pressure in older adults aged 65 and above receiving long-term care services in South Korea and to identify factors influencing pressure levels. The goal was to develop effective strategies for pressure injury prevention in real-world community settings.

### 2.2. Participants

Participants were community-dwelling older adults aged 65 years or older who were receiving long-term care services and were capable of maintaining a supine position for at least 20 min. Inclusion criteria required individuals to be free of acute musculoskeletal or neurological conditions that would interfere with posture maintenance or pressure measurement. Individuals with communication difficulties (e.g., advanced dementia or aphasia), unstable vital signs, skin damage in the sacral area, or inability to lie still were excluded. All participants voluntarily consented to the study. The required sample size was calculated using G*Power 3.1.9.7. With an effect size of 0.15, significance level (α) of 0.05, power of 0.85, and 12 independent variables, the minimum sample size was estimated to be 175. Considering a dropout rate of 20%, a total of 210 participants were recruited.

### 2.3. Data Collection and Measurement Instruments

Data was collected using a structured case report form, which included personal, health-related, and environmental factors. Personal characteristics included sex, age, height, weight, mobility, bowel/bladder function, and nutritional status. Bowel and bladder function were assessed by direct observation and a caregiver report using a structured checklist. Hemoglobin was measured via capillary blood sampling using a portable point-of-care testing device (CERA-CHEK™ Hb Plus), and body weight was measured using a calibrated digital scale. These physiological variables were included to evaluate changes in hemodynamic status before and after pressure redistribution. Cognitive status, medication use, and primary caregiver status were also recorded.

Health-related conditions included chronic diseases, presence of pain or catheters, fall risk, and pressure injury risk. The Braden Scale was used for pressure injury risk (score < 18 = high risk), and the Huhn Scale for fall risk (score ≥ 10 = high risk).

Environmental factors such as room temperature and humidity were also documented at the time of measurement, although they were not included in the main statistical analysis.

### 2.4. Sacral Interface Pressure Measurement

Interface pressure at the sacral area was measured using a portable Palm Q7 device (Cape Co., Ltd., Yokosuka, Japan) while participants were in a standardized supine position. The device includes five pressure sensors in a 10 cm square layout and records values from 0 to 200 mmHg.

The supine position was defined as lying flat while facing upward, with legs extended and separated by approximately 10 cm, and both hands resting on the abdomen. The pressure sensor pad was positioned at the intersection of the bilateral iliac crests, spinal column, and sacrum.

Measurements were conducted twice for each participant: once on a general mattress and once on a pressure-redistributing mattress. Each session lasted 20 min. Prior to and after each session, participants’ health status, hemoglobin, and body weight were assessed as previously described in Section 2.3.

To maintain ethical standards and participant comfort, the pressure sensor was placed over light clothing rather than directly on the skin. This method aligns with the manufacturer-provided instructional video and is consistent with the procedures employed in previous studies using the same device [9].

To ensure consistent measurement, the research team received standardized training using a manufacturer-provided instructional video and a Korean-language checklist. To assess inter-rater reliability, two trained raters independently measured interface pressure at the sacrum in 30 older adults using the Palm Q7, with a 5 min interval between measurements. The single-measure Intraclass Correlation Coefficient (ICC) was 0.938 (95% CI: 0.779–0.976), indicating excellent reliability.

### 2.5. Data Analysis

Data were analyzed using SPSS version 26.0. Descriptive statistics were used to summarize participant characteristics and sacral interface pressure levels. Independent *t*-tests and one-way ANOVA were conducted to compare pressure differences across subgroups. Logistic regression analysis was performed to identify factors associated with high sacral interface pressure. Predictor variables included demographic characteristics (age, sex, BMI) and clinical factors such as Braden Scale score, Huhn fall risk score, cognitive status, bowel and bladder function, and mattress use.

**Figure 1:**
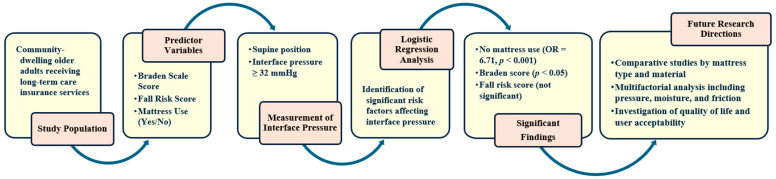
Overview of study design, pressure measurement procedure, and analytical framework.

The Braden Scale was selected as the primary risk assessment tool for pressure injuries in this study. This decision was based on the community-based setting of the research, as recent studies have shown that the Braden Scale demonstrates better predictive validity and clinical applicability than the Waterlow Scale in similar environments [20,21]. The overall research process, including study population, predictor variables, pressure measurement procedures, and analytical framework, is illustrated in Figure 1.

### 2.6. Ethical Considerations

This study was approved by the Institutional Review Board of Chungbuk National University (IRB No. CBNU-2025-A-0001). All participants were informed of the study’s purpose, procedures, and pressure measurement protocol at least one day in advance. Participation was voluntary, and subjects were allowed to withdraw at any time without penalty. Participants were also instructed to report any discomfort during measurement, which would prompt immediate discontinuation. For ethical reasons, the pressure sensor was attached over clothing rather than directly to the skin. To ensure privacy, each participant was assigned a unique code, and all data were anonymized and securely stored in a password-protected computer accessible only to the research team.

Generative AI was not used in study design, data analysis, or interpretation. Only language editing assistance was applied. The dataset generated during the current study is available from the corresponding author on reasonable request.

## 3. Results

### 3.1. General Characteristics of the Participants

A total of 210 participants were included in the study, comprising 137 males (65.2%) and 73 females (34.8%), with a mean age of 82.30 ± 7.56 years. The majority of participants (59.7%) were aged 81 years or older. Regarding mobility, 109 participants (51.9%) were able to ambulate independently, 51 (24.3%) were partially bedridden, and 50 (23.8%) were completely bedridden. The average body mass index (BMI) was 21.59 ± 3.48 kg/m^2^, with 150 participants (72.5%) falling within the normal BMI range (18.5–25.5 kg/m^2^).

In terms of incontinence care, 54 participants (25.7%) used diapers for bowel incontinence and 50 (23.8%) for urinary incontinence, while 38 participants (18.1%) had indwelling urinary catheters. Nutritional status was reported as good in 128 participants (62.1%) and very poor in 7 (3.4%). The mean hemoglobin level was 12.32 ± 1.61 g/dL, and 198 participants (95.2%) had levels below 10 g/dL. The mean oxygen saturation (SpO_2_) was 96.43 ± 2.48%, with 90 participants (60.4%) recording levels of 97% or higher.

Pressure injury staging revealed that 159 participants (75.7%) were classified as stage 0, 23 (11.0%) as stage 1, 18 (8.6%) as stage 2, 7 (3.3%) as stage 3, and 2 (1.0%) as stage 4. One participant (0.5%) was classified as unstageable.

Fall risk assessment showed that 135 participants (64.3%) were categorized as high-risk (score ≥ 11), with a mean fall risk score of 11.98 ± 8.66. According to the Braden Scale, 20 participants (9.5%) were classified as high-risk (≤9 points) and 92 (43.8%) as moderate-risk (10–17 points), with a mean score of 16.55 ± 4.81.

Regarding mattress use, 137 participants (65.2%) used either an air or foam pressure-relieving mattress, while 73 participants (34.8%) did not (Table 1).

### 3.2. Differences in Sacral Interface Pressure According to General Characteristics

Based on prior research [22], sacral interface pressure was classified using a threshold of 32 mmHg. Statistically significant differences were observed in sacral interface pressure according to fall risk (χ^2^ = 9.29, *p* = 0.010), Braden Scale scores (χ^2^ = 11.18, *p* = 0.004), and mattress use (χ^2^ = 8.17, *p* = 0.004).

Among participants at high risk of falls (≥11 points), 80.0% (*n* = 108) exhibited sacral interface pressure ≥32 mmHg, compared to 60.7% (*n* = 37) in the low-risk group (≤4 points), suggesting that individuals at greater fall risk are more likely to experience elevated interface pressure.

Similarly, among participants with sacral interface pressure ≥32 mmHg, 85.9% (*n* = 79) were classified as moderate risk (10–17 points) based on the Braden Scale, while 67.3% (*n* = 66) were classified as having normal risk (≥18 points). This indicates that individuals with reduced mobility, sensory perception, moisture control, or nutritional status—as assessed by the Braden Scale—may be more prone to increased sacral pressure.

Regarding mattress use, 86.6% (*n* = 58) of participants who did not use a pressure-relieving mattress showed sacral interface pressure ≥32 mmHg, highlighting the potential effectiveness of pressure redistribution mattresses.

No statistically significant differences in sacral interface pressure were found with respect to sex, age, bedridden status, BMI, nutritional status, hemoglobin level, oxygen saturation, or urinary and defecation care variables (Table 2).

### 3.3. Factors Influencing Sacral Interface Pressure

A multiple logistic regression analysis was conducted using the variables that were statistically significant in the univariate analysis (*p* < 0.05), including fall risk score, Braden Scale score, and mattress use, to identify the risk factors associated with elevated sacral interface pressure. The regression model was statistically significant (χ^2^ = 35.70, *p* < 0.001), with a Cox and Snell R-squared value of 0.16 and a Nagelkerke R-squared value of 0.23, indicating moderate explanatory power (Table 3). The analysis revealed that non-use of a pressure-relieving mattress was the most significant predictor of elevated interface pressure. Participants who did not use a mattress were 6.71 times more likely to exhibit sacral interface pressure levels ≥ 32 mmHg compared to those who used a mattress (OR = 6.71, *p* < 0.001).

Additionally, Braden Scale scores were found to have a statistically significant association with interface pressure. Participants in the moderate-risk group (10–17 points) had significantly higher odds of elevated sacral interface pressure compared to those in the normal group (≥18 points), indicating that impaired skin integrity, reduced mobility, and related risk factors may contribute to increased interface pressure in older adults.

## 4. Discussion

### 4.1. Summary of Main Findings

**Figure 2:**
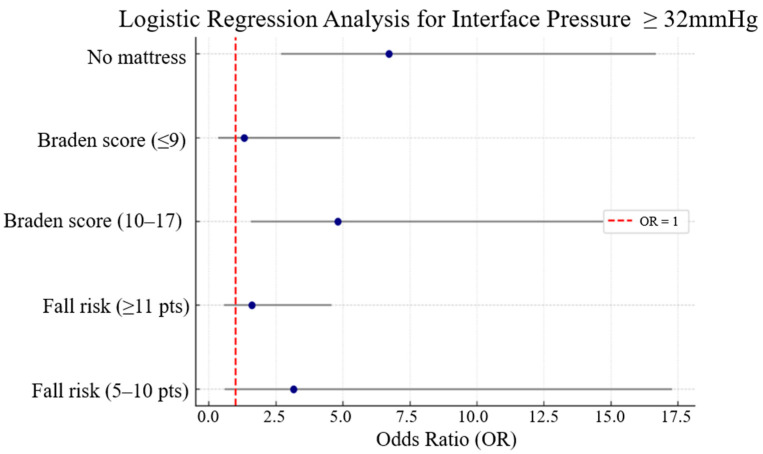
Forest plot of logistic regression analysis for factors influencing interface pressure (≥32 mmHg) in the supine position.

This study analyzed the factors influencing sacral interface pressure in community-dwelling older adults. The results indicated that the non-use of pressure-relieving mattresses was the most significant predictor of elevated interface pressure (OR = 6.71, *p* < 0.001), followed by Braden Scale scores, which also showed a statistically significant association (Figure 2). In contrast, Huhn Scale scores did not show a meaningful relationship, suggesting that skin condition and support surface characteristics may have a stronger impact on sacral interface pressure than mobility-related risk factors.

### 4.2. Interpretation in the Context of Prior Research

These findings are consistent with previous studies. For example, Kawabata et al. [9] reported a relationship between internal air pressure in mattresses and interface pressure distribution, while Matsuo et al. [13] proposed a pressure redistribution model emphasizing the clinical relevance of Braden Scale components. Similarly, Ho et al. [23] demonstrated the effectiveness of real-time pressure mapping systems in reducing interface pressure among high-risk individuals.

### 4.3. Methodological Considerations

Ethical considerations limited the direct measurement of interface pressure on the skin; instead, sensors were placed over participants’ clothing. This indirect approach may have introduced some inaccuracy in absolute pressure values; thus, the findings should be interpreted in terms of relative differences. Future research should consider additional physiological and environmental variables—such as skin friction, moisture, and temperature—to better understand the complex dynamics of interface pressure.

### 4.4. Practical and Technological Implications

This study was conducted not in a hospital setting but in real-world community environments. Therefore, various uncontrolled external factors may have influenced sacral interface pressure. Prior research has shown that when family members serve as primary caregivers, insufficient knowledge, attitudes, and practices can hinder effective pressure injury prevention [24]. This may help explain the elevated pressure observed even among participants classified as “moderate risk” by the Braden Scale. These findings highlight the importance of tailored nursing interventions that reflect actual living conditions.

Specifically, smart mattresses and pressure-redistributing surfaces have been shown to effectively reduce sacral interface pressure in high-risk older adults [25,26]. However, such high-cost equipment is often difficult to implement widely in community settings due to financial and structural limitations. In this context, prioritizing mattress provision for high-risk individuals may improve cost-effectiveness and system efficiency [27]. Thus, policy approaches should go beyond equipment distribution to include caregiver education, low-cost environmental modifications, and the development of integrated community-based support systems. In addition, effective risk assessment for pressure injury in older adults should be led by a multidisciplinary team (MDT), incorporating not only the Braden Scale but also comprehensive evaluations of nutritional status and physical function [28]. This team-based, holistic assessment approach can better identify those at highest risk and facilitate the delivery of targeted, practical interventions tailored to real-world community environments.

Future strategies should focus on developing affordable, user-friendly technologies that can be selectively applied to high-risk groups and integrated into community health systems. Ultimately, pressure injury prevention should evolve into a sustainable, user-centered nursing model that integrates clinical evaluation, technology, and community-based care.

### 4.5. Limitations

This study has several limitations.

First, for ethical considerations and to protect personal privacy, interface pressure was measured by placing the sensor over clothing rather than directly on the skin. Although the study focused on relative differences between conditions rather than absolute values, this indirect measurement method may have affected the accuracy of pressure readings.

Second, due to the cross-sectional design of this study, it is difficult to establish clear causal relationships between the identified influencing factors and sacral interface pressure.

Third, participants were recruited from a single community health center in an urban area, which limits the generalizability of the findings to older adults living in rural settings or long-term care facilities.

Fourth, physiological factors that may affect interface pressure—such as skin moisture, temperature, and friction—were not measured or included in the analysis.

Future research should consider direct pressure measurements on the skin, employ longitudinal study designs, and include a broader range of physiological and environmental variables to deepen understanding of pressure dynamics among community-dwelling older adults.

## 5. Conclusions

This study confirmed that the non-use of pressure-relieving mattresses and lower Braden Scale scores were significantly associated with increased sacral interface pressure among community-dwelling older adults. These results suggest that reliance solely on clinical assessment is insufficient, and more targeted preventive strategies are needed. Improving access to affordable pressure-relieving equipment and strengthening caregiver education in the home are essential. In addition, the development of smart pressure monitoring technologies and community-based nursing interventions should be prioritized to reduce risks among the elderly. Furthermore, the assessment of older adults at risk of pressure injury should be led by a multidisciplinary team (MDT) and include comprehensive evaluations of nutritional status and physical function, in addition to the Braden Scale. Future research should further refine and validate these approaches in various real-world settings.

## Figures and Tables

**Table 1:** General characteristics of participants.

Variables	Categories		*n*	%	Mean ± SD
Gender	Male		137	65.2	
	Female		73	34.8	
Age	≤70		14	6.8	82.30 ± 7.56
	71–80		69	33.5	
	≥81		123	59.7	
Bedridden status	Fully		50	23.8	
	Partially		51	24.3	
	Not bedridden		109	51.9	
BMI (kg/m^2^)	<18.5		34	16.4	21.59 ± 3.48
	18.5–25.5		150	72.5	
	≥25.5		23	11.1	
Defecation	No diaper		156	74.3	
	With diaper		54	25.7	
Urination	No diaper		160	76.2	
	With diaper		50	23.8	
Urinary catheter	None		172	81.9	
	Present		38	18.1	
Nutritional status	Good		128	62.1	
	Poor		71	34.5	
	Very poor		7	3.4	
Hemoglobin (Hb,g/dL)	≥10		10	4.8	12.32 ± 1.61
	<10		198	95.2	
SpO_2_ (%)	≥97		90	60.4	96.43 ± 2.48
	<97		59	39.6	
Pressure injury	No		159	75.7	
	Yes	Stage 1	23	11	
		Stage 2	18	8.6	
		Stage 3	7	3.3	
		Stage 4	2	1	
		Unstageable	1	0.5	
Huhn Scale score for fall risk	Low (≤4)		61	29	11.98 ± 8.66
	Moderate (5–10)		14	6.7	
	High (≥11)		135	64.3	
Braden Scale score	Normal (≥18)		98	46.7	16.55 ± 4.81
	Moderate risk (10–17)		92	43.8	
	High risk (≤9)		20	9.5	
Mattress use	No		73	34.8	
	Yes		137	65.2	

**Table 2:** Differences in sacral interface pressure according to general characteristics (*n* = 210).

Variables	Categories		Interface Pressure *n* (%)		χ^2^/t/Z	*p*
			<32 mmHg	≥32 mmHg		
Gender	Male		37 (27.0)	100 (73.0)	0.65	0.419
	Female		16 (21.9)	57 (78.1)		
Age	≤70		2 (14.3)	12 (85.7)	0.96	0.620
	71–80		18 (26.1)	51 (73.9)		
	≥81		32 (26.0)	91 (74.0)		
Bedridden status	Fully		15 (30.0)	35 (70.0)	4.78	0.092
	Partially		7 (13.7)	44 (86.3)		
	Not bedridden		31 (28.4)	78 (71.6)		
BMI	<18.5		10 (29.4)	24 (70.6)	0.49	0.783
	18.5–25.5		37 (24.7)	113 (75.3)		
	≥25.5		5 (21.7)	18 (78.3)		
Defecation	No diaper		41 (26.3)	115 (73.7)	0.35	0.554
	With diaper		12 (22.2)	42 (77.8)		
Urination	No diaper		42 (26.3)	118 (73.8)	0.37	0.546
	With diaper		11 (22.0)	39 (78.0)		
Indwelling catheter	No		44 (25.6)	128 (74.4)	0.06	0.807
	Yes		9 (23.7)	29 (76.3)		
Nutritional status	Good		39 (30.5)	89 (69.5)	4.92	0.086
	Poor		12 (16.9)	59 (83.1)		
	Severely poor		1 (14.3)	6 (85.7)		
Hemoglobin (Hb,g/dL)	≥10 g/dL		5 (50.0)	5 (50.0)	3.50	0.061
	<10 g/dL		47 (23.7)	151 (76.3)		
SpO_2_ (%)	≥97%		21 (23.3)	69 (76.7)	1.45	0.229
	<97%		9 (15.3)	50 (84.7)		
Pressure injury	No		40 (25.2)	119 (74.8)	4.27	0.511
	Yes	Stage 1	5 (21.7)	18 (78.3)		
		Stage 2	5 (27.8)	13 (72.2)		
		Stage 3	1 (14.3)	6 (85.7)		
		Stage 4	1 (50.0)	1 (50.0)		
		Unstageable	1 (100.0)	0 (0.0)		
Fall risk score	Low (≤4)		24 (39.3)	37 (60.7)	9.29	0.010
	Moderate (5–10)		2 (14.3)	12 (85.7)		
	High (≥11)		27 (20.0)	108 (80.0)		
Huhn Scale score for fall risk	Normal (≥18)		32 (32.7)	66 (67.3)	11.18	0.004
	Moderate risk (10–17)		13 (14.1)	79 (85.9)		
	High risk (≤9)		8 (40.0)	12 (60.0)		
Mattress use	No		9 (13.4)	58 (86.6)	8.17	0.004
	Yes		44 (32.1)	93 (67.9)		

**Table 3:** Logistic regression analysis of multiple factors influencing interface pressure.

Variable	Categories	B	SE	*p*	OR	95% CI	
						Lower	Upper
Fall risk score	Low risk (≤4 points, reference)						
	Moderate risk (5–10 points)	1.15	0.87	0.184	3.16	0.58	17.27
	High risk (≥11 points)	0.47	0.54	0.381	1.6	0.56	4.57
Braden Scale score	Normal (≥18, reference)						
	Moderate risk (10–17)	1.57	0.57	0.006	4.8	1.57	14.7
	High risk (≤9)	0.27	0.67	0.692	1.31	0.35	4.9
Mattress use	Yes (reference)						
	No	1.9	0.46	<0.001	6.71	2.7	16.67
Nagelkerke R2 = 0.23; Cox and Snell R2 = 0.16; χ^2^/*p*-value = 35.70/<0.001							

SE = standard error; OR = odds ratio; CI = confidence interval.

## Data Availability

Data are contained within the article.
